# Establishment and application of isothermal amplification techniques for the detection of heat-stable I enterotoxin of enterotoxigenic *Escherichia coli*

**DOI:** 10.1371/journal.pone.0230881

**Published:** 2020-04-21

**Authors:** Junjun Zhai, Zhang Yan, Feng Ping, Qu Lei, Xuelong Chen, Yanping Qi

**Affiliations:** 1 Shaanxi Province Engineering & Technology Research Center of Shanbei Cashmere Goats, Yulin University, Yulin, Shanxi, China; 2 College of Life Science, Yulin University, Yulin, Shanxi, China; 3 College of Animal Science, Anhui Science and Technology University, Fengyang, Anhui, China; Xinyang Normal University, CHINA

## Abstract

The aim of this study was to establish isothermal multiple self-matching initiated amplification (IMSA) and cross-priming amplification (CPA) methods to detect heat-stable I enterotoxin (STa)-producing enterotoxigenic *Escherichia coli* (ETEC). To avoid cross-contamination of aerosols, a closed independent isothermal amplification tube (IAT) was used to perform the assays. Optimal amplification conditions for IMSA and CPA were selected for specificity and sensitivity, respectively, and for clinical relevance. Both IMSA and CPA assays could specifically recognize all 3-STa positive strains in which they fluoresced green under UV light, but not in the 11 non-STa strains. The results of the sensitivity analysis indicated that the detection limit of the IMSA assay was 1.5 ×10^2^ CFU, comparable to real-time PCR, but 10-fold more sensitive than CPA and LAMP. Further evaluation of the detection methods of swine diarrhea samples demonstrated that both could successfully identify the DNA of STa-producing ETEC in clinical specimens, consistent with LAMP and qPCR methods. The results demonstrated that the IMSA and CPA methods had high specificity and sensitivity with rapid detection of ETEC, so having great potential in clinical applications.

## Introduction

Infection by enterotoxigenic *Escherichia coli* (ETEC) is a major cause of acute diarrhea in animals and humans and plays an important role in the etiology of food-borne and water-borne outbreaks, especially in developing countries [1–3]. Diarrhea caused by ETEC brings great economic loss on an annual basis to the global animal industry, among which the most conspicuous manifestation is disease in and death of suckling and weaning piglets [4, 5]. The factors that cause the foremost virulence in ETEC strains include adhesins and/or enterotoxins, allowing the bacteria to colonize the epithelial cells of the small intestines of animals after which heat-labile enterotoxin (LT, divided into LT-Ⅰ and LT-Ⅱ) and/or heat-stable enterotoxin (ST, divided into STa and STb) is released, inducing intestinal epithelial cells to cause electrolyte disorder, fluid retention and diarrhea [6, 7]. Among these factors, heat-stable I enterotoxin has been reported to be isolated from human and animal samples, and food and water over the past few decades. Epidemiological data suggest that more than one-quarter of cases of porcine ETEC diarrhea and over two-thirds of human ETEC diarrhea are caused by STa-producing ETEC strains [6, 8, 9]. Therefore, regular monitoring of STa prevalence in swineries is an effective method of preventing ETEC infection.

Diagnosis of ETEC infection currently relies on the differentiation of phenotype of pathogenic strains from normal nonpathogenic flora via bioassays or immunoassays for toxins and fimbriae [10]. A series of diagnostic methods have been established that identify STa antigen, including the suckling-mouse assay, enzyme-linked immunosorbent assay (ELISA) and polymerase chain reaction (PCR) including multiple PCR and real-time PCR assays [11, 12]. Although some methods can be used as a “gold standard”, the assay results demonstrating high reliability, they have a number of drawbacks such as requiring complex operation, are time-consuming, with high cost, false-positivity and requiring considerable operator consistency, *etc*., and thus cannot meet the need for early field diagnosis [13].

In recent years, a number of novel isothermal amplification technologies (IATs) have been successively established and used for the detection of pathogenic microorganisms. They are able to replace or compensate for the shortcomings of traditional detection methods. Loop-mediated isothermal amplification (LAMP) is among the most widely-used isothermal amplification techniques which has the same sensitivity and specificity as real-time PCR, and which has been used for the clinical detection of a variety of pathogenic microorganisms [14, 15]. The method only requires four to six primers that recognize six to eight regions of Bst, Bsm or GspSSD polymerase enzyme in the target DNA, and which exhibits strand displacement activity [16]. Furthermore, it only requires a simple heating device to detect the pathogens within 2 h which allows for rapid detection of a large quantity of target DNA using various formats, such as trapezoidal strips formed on agarose gel electrophoresis or direct combination with fluorescent dyes observed directly under visible light. LAMP has emerged as a powerful tool for diagnosis and has been successfully developed to detect ETEC strains [17]. Isothermal multiple-self-matching initiated amplification (IMSA) is a novel technique whose mechanism was first described by Ding *et al*., in which three pairs of primers, including a pair of stem primers (SteF and SteR) and two pairs of hybrid nested primers (outer forward and reverse, and inner forward and reverse hybrid-primers). The primers are able to specifically recognize distinct corresponding regions of the target DNA and can typically generate multiple self-matching structures (SMS) similar to a dumbbell, which single-stranded DNA amplicons generate from the hybrid primers. This technology does not require complex instruments and the detection of target DNA sequences can be completed within 1–2 hours using isothermal conditions, for which the method is potentially rapid and simple allowing practical diagnosis of a specific disease [18, 19]. Cross-priming amplification (CPA) was invented by Fang *et al*. which uses a mechanism similar to LAMP with 4–8 nucleotide primers complementary to the DNA template and using a detection procedure achieved using isothermal conditions within 1 h [20, 21]. During CPA amplification, double-stranded DNA can be separated at constant temperature, after which the products form hairpin structures catalyzed by polymerase with strand-displacement activity and generate products with interval tandem repeats [22]. Furthermore, the products can be detected visually after the addition of any fluorescent dye (such as SYBR Green I), as is the case for any double-stranded DNA in the majority isothermal amplification techniques. Multiple reports have been published indicating that CPA technology has been successfully used as a specific and sensitive method for the detection of *Mycobacterium tuberculosis*, *Enterobacter sakazakii* and heat labile enterotoxin of *Escherichia coli etc*. [20, 23, 24].

The objective of the current study was to develop and compare two rapid, sensitive, cost-effective isothermal amplification methods (IMSA and CPA) for the identification of STa-producing ETEC strains isolated from diarrhea specimens, to ascertain their potential in monitoring applications in developing countries.

## Materials and methods

### 2.1 Bacterial strains and DNA extraction

A total 14 bacterial strains, including 8 strains of *Escherichia coli* (3 positive for STa and 5 non-STa^+^ strains) and 6 that were not *Escherichia coli* were used for the IMSA and CPA assays (Table 1). All reference strains were cultured on nutrient agar plates (Sigma-Aldrich, St. Louis, MO, USA) at 37°C overnight. A individual colony selected from each plate was inoculated into 5 mL LB medium and incubated at 37°C for 18 h in a constant temperature shaking table (LPH-200D, labCAN Instrument Equipment Co,. Ltd, Jiangsu, China). All bacterial genomic DNA was then extracted from each strain using a bacteria DNA extraction kit (Invitrogen, Waltham, MA). *Escherichia coli* C83920 was used for standardization, optimization and to determine the sensitivity of the IMSA and CPA assays.

**Table 1:** Bacterial strains used in this study.

Bacterial strain	Source	CPA	IMSA	LAMP
Enterotoxigenic *E*. *coli* ATCC 35401 (STa^+^, LT-I^+^)	ATCC^a^	+	+	+
Enterotoxigenic *E*. *coli* C83920 (STa^+^)	IVDC^b^	+	+	+
Enterotoxigenic *E*. *coli* SD-12-01 (STa^+^)	Clinical-isolate^c^	+	+	+
Enterotoxigenic *E*. *coli* C83915 (LT-I^+^)	IVDC	-	-	-
Verotoxigenic *E*. *coli* O157: H7 (Stx1+, Stx2^+^)	IVDC	-	-	-
Enterotoxigenic *E*. *coli* C44498 (Stx2e^+^)	IVDC	-	-	-
Enterotoxigenic SD-02-01 (STb^+^)	Clinical-isolate	-	-	-
Enterotoxigenic SD-17-03(LT-I^+^)	Clinical-isolate	-	-	-
*Aeromonas hydrophila* ATCC 7966	ATCC	-	-	-
*Listeria monocytogenes* ATCC 19115	ATCC	-	-	-
*Salmonella enteritidis* ATCC 13076	ATCC	-	-	-
*Staphylococcus aureus* ATCC 25923	ATCC	-	-	-
*Yersinia enterocolitica* ATCC 23715	ATCC	-	-	-
*Vibrio parahaemolyticus* ATCC 27519	ATCC	-	-	-

^a^ American Type Culture Collection.

^b^ China Institute of Veterinary Drug Control.

^c^ Clinical isolates were preserved in our lab. + positive result.—negative result.

#### Primers

**Fig 1:**
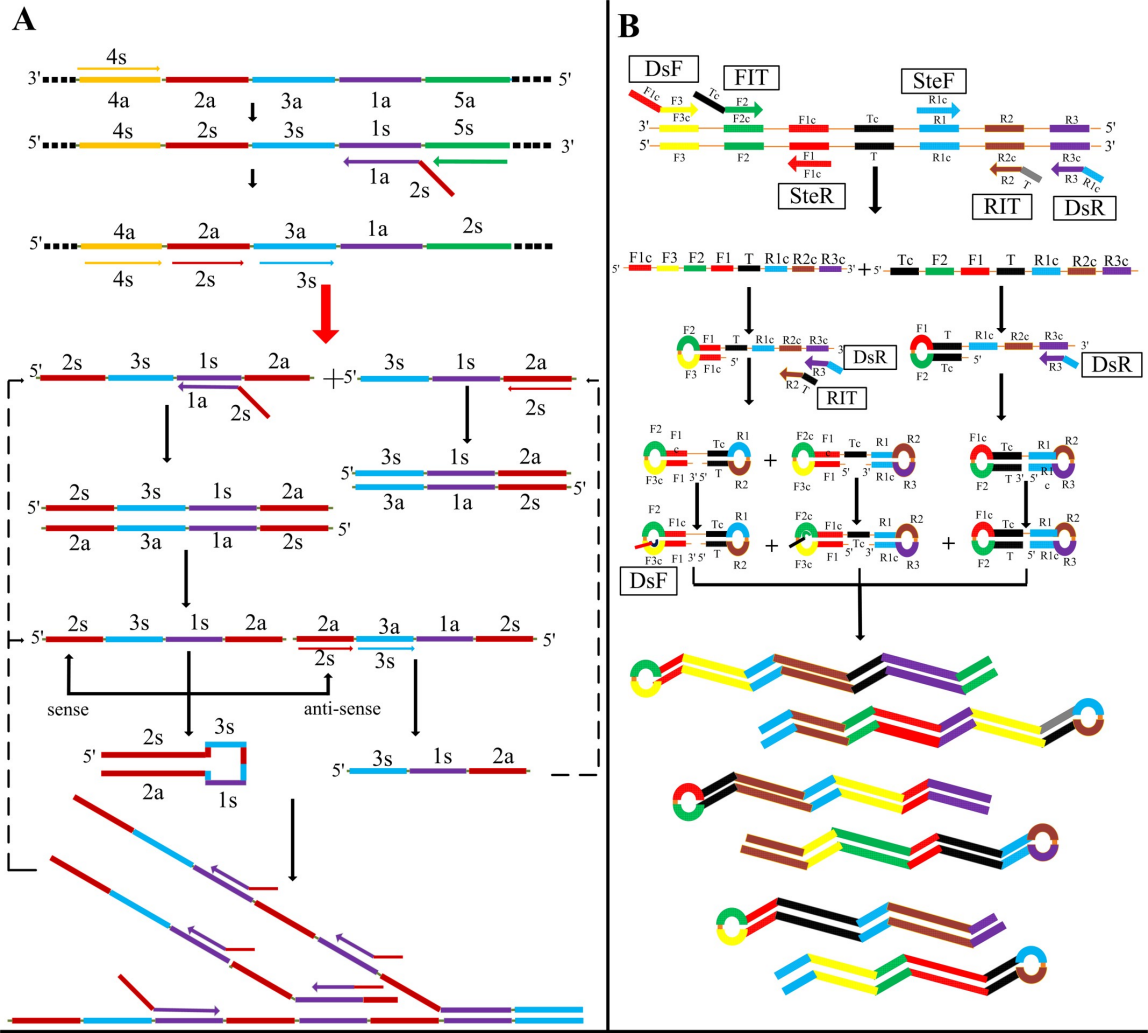
Amplification diagrammatic sketch for CPA and IMSA. A. Ideographic of the CPA assay. B. Ideographic of the IMSA assay.

Specific primers for CPA and IMSA were designed based on sequences of the STa gene available from the GenBank database (No: M25607.1), as shown in Fig 1. The design principle of the CPA primers followed Fang *et al*., in which five primers termed 1s (cross primer), 2a/3a (displacement primers) and 4s/5a (detector primers) that targeted five distinct regions of the STa sequence were designed using Primer Premier 5.0 software (Premier Biosoft International, Palo Alto, CA). A set of IMSA primers consisting of two stem primers (SteF/SteR), two outer primers (DsF/DsR) and two inner primers (FIT/RIT) were also designed. The specific primers for the LAMP assay have been described in a previous report, in addition to the principle of primer design, published by Yano *et al*. [25].

In addition, a pair of fluorescent real-time PCR primers was included as a standard reference to allow calculation of sensitivity and relevance for clinical assays [26]. All the sequences of oligonucleotide primers designed here were compared with the NCBI to verify their specificity, as shown in Table 2. The primers used in this study were synthesized by Sangon Co., Ltd. (Shanghai, China).

**Table 2:** Primers for LAMP, CPA, IMSA and real-time PCR assays.

Item	Primer Name	Primer Sequence (5'-3')
LAMP Primers	F3	CTCAGGATGCTAAACCAGT
	B3	CAGAACAAATATAAAGGGAACTGTT
	FIP	TCATGCTTTCAGGACCACTTTTATTGAGTCTTCAAAAGAAAAAATCACACT
	BIP	AGTAGCAATTACTGCTGTGAATTGTCCCTTTATATTATTAATAGCACCCG
	LB	GTTGTAATCCTGCTTGT
CPA Primers	1s	TGTTAATTGTCTGGCTATCTGGTTGGATAAAACCATCACTTAATGTGA
	2a	TGTTAATTGTCTGGCTATCTGGTT
	3a	CTATCTGGTTTTTTTTACTATCAATATCAC
	4s	GGAGTCATTACCCGAACAA
	5a	ACCTCCCGTCATGTTGTT
IMSA primers	DsF	CACACTTTTTAGTCTCTAATGTAATTTTCTCTTTATTTCTGTATTATCTTTCCCC
	DsR	CTGTGAACTTTGTTGTAATCCTGCC-GGGAATCAAAATAAAGATTCCC
	FIT	CATATTTTCTGATTTTTTTTCACTGTTGTAGTCAGTCAACTGAATCACTTGAC
	RIT	CAACAGTGAAAAAAAATCAGAAAATATGGCTTTTTAATAACATGGAGCAC
	SteF	CTGTGAACTTTGTTGTAATCCTGCC
	SteR	CACACTTTTTAGTCTCTAATGTAATTTTCTC
Q-PCR primers	Forward	CTGTATTATCTTTCCCCTCT
	Reverse	TTTAATAACATGGAGCACAG

### Assay reactions

To avoid cross-contamination of aerosols, an isothermal amplification tube (IAT, Guangzhou Hua-feng Biological Co., Ltd, Guangdong, China) was used as a separate containment control for evaluating the results (visualization using ultraviolet light). The CPA assay was conducted in a reaction mixture of 30 μL total volume containing 6 μL 1s (10 mM), 1.5 μL 2a/3a (10 mM), 0.5 μL 4s/5a (10 mM), 2.5 μL (1.0 mM) dNTPs (Takara Bio, Dalian, CN), 3 μL 10 × Thermol-Pol reaction buffer, 1.5 μL Bst DNA polymerase large fragment (New England Biolabs, Ipswich, MA, USA), 3 μL (1 mM) MgCl_2_ (Sigma-Aldrich, St. Louis, MO, USA), 2 μL (0.8 M) betaine (Sigma-Aldrich) and 2 μL of an appropriate concentration of DNA template (*E*. *coli* C83920). The CPA reaction was then performed at 63°C for 1 h and then terminated by incubation at 85°C for 3 min. For the IMSA assay, the reaction was also performed in a 30 μL mixture, which contained 1 μL (10 mM) outer primers DsF/DsR, 2.5 μL (10 mM) FIT/RIT and SteF/SteR, respectively, 3 μL 10 × Thermol-Pol reaction buffer, 2 μL (16 U) Bst DNA polymerase large fragment, 3 μL (1 mM) MgCl_2_, 2 μL (0.8 M) betaine, 2.5 μL (1.0 mM) dNTPs and 2 μL of target DNA, the reaction system supplemented with sterilized deionized water. Amplification was accomplished at 63°C for 60 min after which the mixture was heated to 85°C for 5 min to terminate the reaction. The LAMP and real-time PCR assays followed the initial reaction using conditions confirmed by previous respective studies. Subsequently, the products were separated by electrophoresis on a 1.5% agarose gel within a Tris–acetic acid–EDTA (TAE) buffer after staining with 0.5% ethidium bromide (EB, Sigma).

### Optimization assay

Optimization of the CPA and IMSA assays involved varying the reaction temperature, incubation time and concentration of the reaction mixture (dNTPs, Mg^2+^, betaine and Bst DNA polymerase). Briefly, the incubation temperature was optimized between 61°C ~ 71°C, at intervals of 2°C. The concentration of Mg^2+^ was varied from 1.0 mM to 4.0 mM, that of *Bst*-DNA polymerase from 8 U to 20 U, dNTPs from 1.0 mM—3.0 mM and the concentration of betaine from 0.6 M to 1.4 M. The last parameter optimized was duration of incubation which ranged from 30 min to 120 min. All optimized assays were tested in triplicate and the results recorded using a real-time turbidimeter (Eiken Chemical Co., Ltd, Tokyo, Japan). Subsequently, the amplified products were tested by 2.0% agarose gel electrophoresis.

### Specificity of the CPA and IMSA assays

The specificity of CPA, IMSA, and LAMP assays were evaluated using 14 different standard and isolated strains, consisting of 8 *E*. *coli* and 6 non-*E*. *coli* strains, as listed in Table 1. Sterilized deionized water was used a blank control and all tests were performed in triplicate. Subsequently, the final products were assayed by 2.0% agarose gel electrophoresis and directly visualized using ultraviolet light (blended with SYBR Green I), respectively.

### Sensitivity of the CPA and IMSA assays

To evaluate the sensitivity of the CPA and IMSA assays, total genomic DNA extracted from *E*. *coli* C83920 harboring STa^+^ was measured. A DNA template with an initial concentration of 1.5×10^8^ CFU/ reaction was then processed with 10-fold serial dilutions so that a range from 1.5 × 10^8^ CFU to 1.5 × 10^1^ CFU per reaction was prepared, in order to establish the detection limits of CPA and IMSA analysis. All amplification assays were performed in triplicate by at least two researchers and amplification results compared with those obtained from real-time PCR and LAMP assays. All products were visualized directly by mixing with 1 μL of SYBR Green I dyes (×2000) then assaying by 2.0% agarose gel electrophoresis, respectively.

### Evaluation of CPA and IMSA in clinical samples

In order to determine the validity and reliability of the CPA and IMSA assays for the detection of STa-producing ETEC in clinical samples, a total of 265 swine diarrhea specimens were collected from weaned piglets in the northeast and northwest of China, which were then assayed in accordance with the standards of the *Escherichia coli* identification procedure [6]. Briefly, all samples were collected with sterile swabs and placed into eppendorf tubes, then sent to the laboratory within 24 h. The fecal samples were directly scribed on sterile MacConkey agar plates (Sigma-Aldrich) and incubated at 37°C overnight. *Escherichia coli*-like colonies were selected and sub-cultured on Luria–Bertani (LB) agar plates (Oxoid, Hampshire, UK) for standard biochemical confirmed identification procedures, including citrate utilization, MR positive, VP negative and hydrogen sulfate production, *etc*. The DNA of all confirmed *E*. coli isolates was extracted and stored at -20°C for subsequent CPA and IMSA analysis, the results of which were compared with the real-time PCR assay.

### Ethics statement

All the diarrhea specimens were collected with the permission of the owner of the farms, complying with the Animal Welfare Act (AWA) and following National Institute of Health (NIH) guidelines (NIH Pub. No. 85–23, revised 1996). The protocols were approved and supervised by the Animal Care and Use Committee of Yulin University (Shanxi, Yulin, China) and Anhui Science and Technology University (Anhui, Fengyang, China), respectively.

## Results

### Amplification of the STa by CPA and IMSA assays

**Fig 2:**
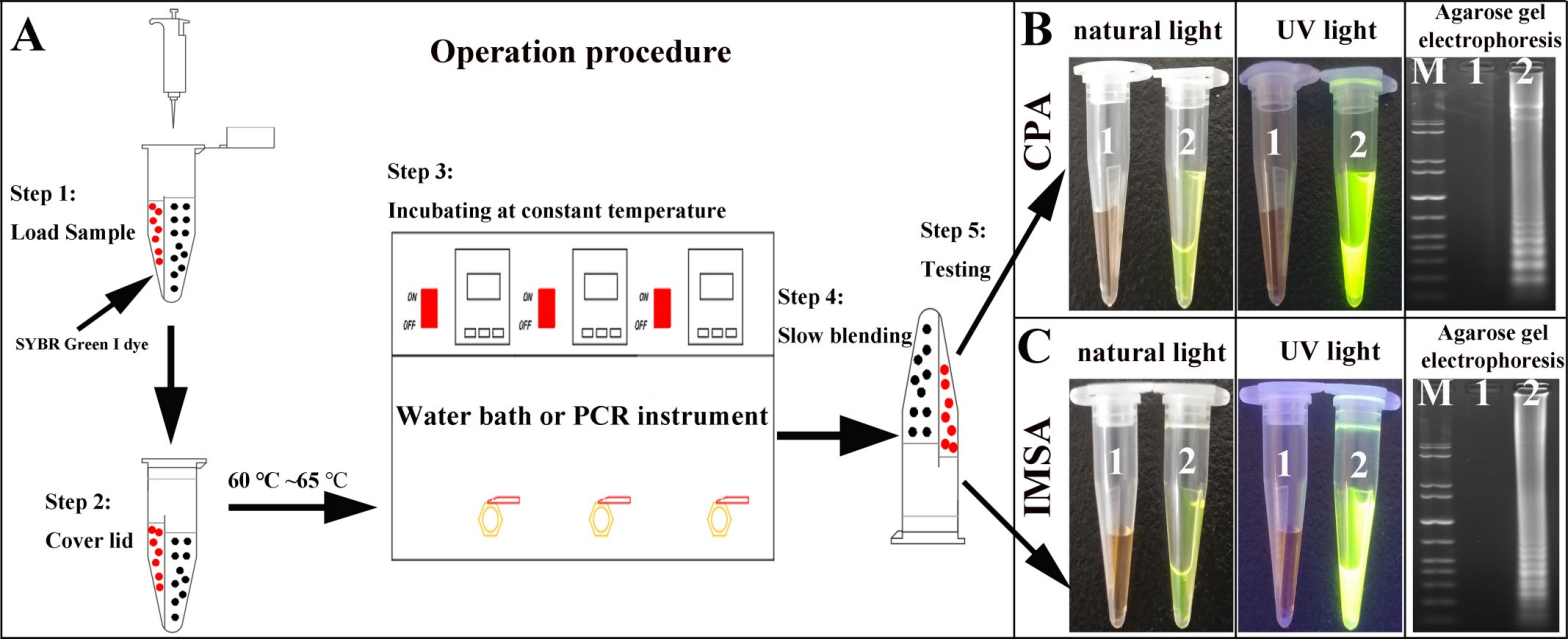
Amplification simulated diagram of the CPA and IMSA assays. A. General reaction simulation diagram. B. Observation by natural and UV light, and agarose gel electrophoresis for CPA, respectively. C. Observation by natural and UV light, and agarose gel electrophoresis for IMSA, respectively. M: standard DNA molecule marker: 1: negative control. 2: amplification of STa gene.

The amplification process is shown conceptually in Fig 2. After amplification, the amplified products of the CPA and IMSA assays were slowly and carefully mixed with SYBR Green I dye on the other side. The final results of the assays could easily be observed with the naked eye when illuminated by ultraviolet light, with green fluorescence due to positive amplification and negative amplification resulting in no fluorescence. In addition, 5 μL of the amplified products of CPA and IMSA were analyzed and detected on 2.0% agarose gel electrophoresis. Similarly, with the LAMP assay, all positive results demonstrated a characteristic irregular ladder-like strip while negative amplification did not exhibit any significant change.

### Optimization assay

**Fig 3:**
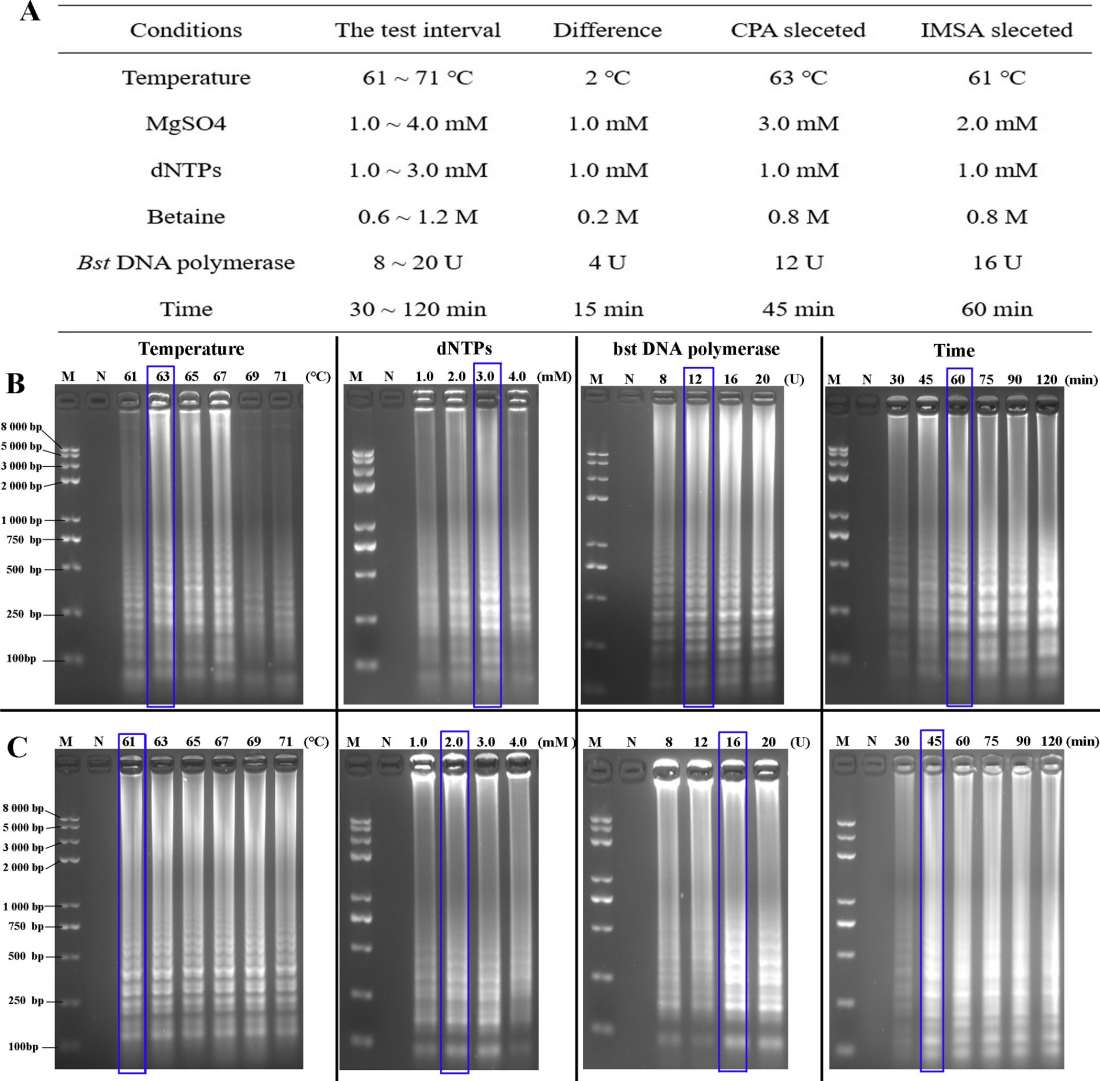
Amplification simulated diagram of the CPA and IMSA assays. A, Conditions and interval selection of assay optimization. B-C. Optimization of the incubation temperature, dNTPs concentration, Bst DNA polymerase concentration and incubation time for the CPA and IMSA assays, respectively. M: Standard DNA molecule marker; N: negative control. (The optimization items, corresponding conditions and units are marked in the figure, the best conditions highlighted with blue squares.).

The success of isothermal amplification depends on the specificity of the design of primers, in addition to other factors inherent in the system. A real-time turbidimeter was used for monitoring the amplification process in each IAT in order that the optimal conditions could be selected (Fig 3). For the CPA assay, optimization of temperature indicated that 63°C was optimal within the range 61°C to 71°C. Optimal duration of incubation was 60 min after testing from 15 min to 120 min. Bst DNA polymerase, dNTPs, Mg^2+^ and betaine concentrations used in the CPA assay of 12 U/ tube, 3.0 mM, 1.0 mM and 0.8 M, respectively, were found to result in the greatest intensity of fluorescence. For the IMSA assay, efficiency of cost per assay was the aim. For procedure requiring the same effect, the lowest cost option should therefore be selected. Thus, the final optimization conditions were as follows: incubation for 45 min at 61°C, 2.0 mM dNTPs, 1.0 mM Mg^2+^, 0.8 M betaine and 16 U/tube of Bst DNA polymerase.

### Specificity of the CPA and IMSA assays

**Fig 4:**
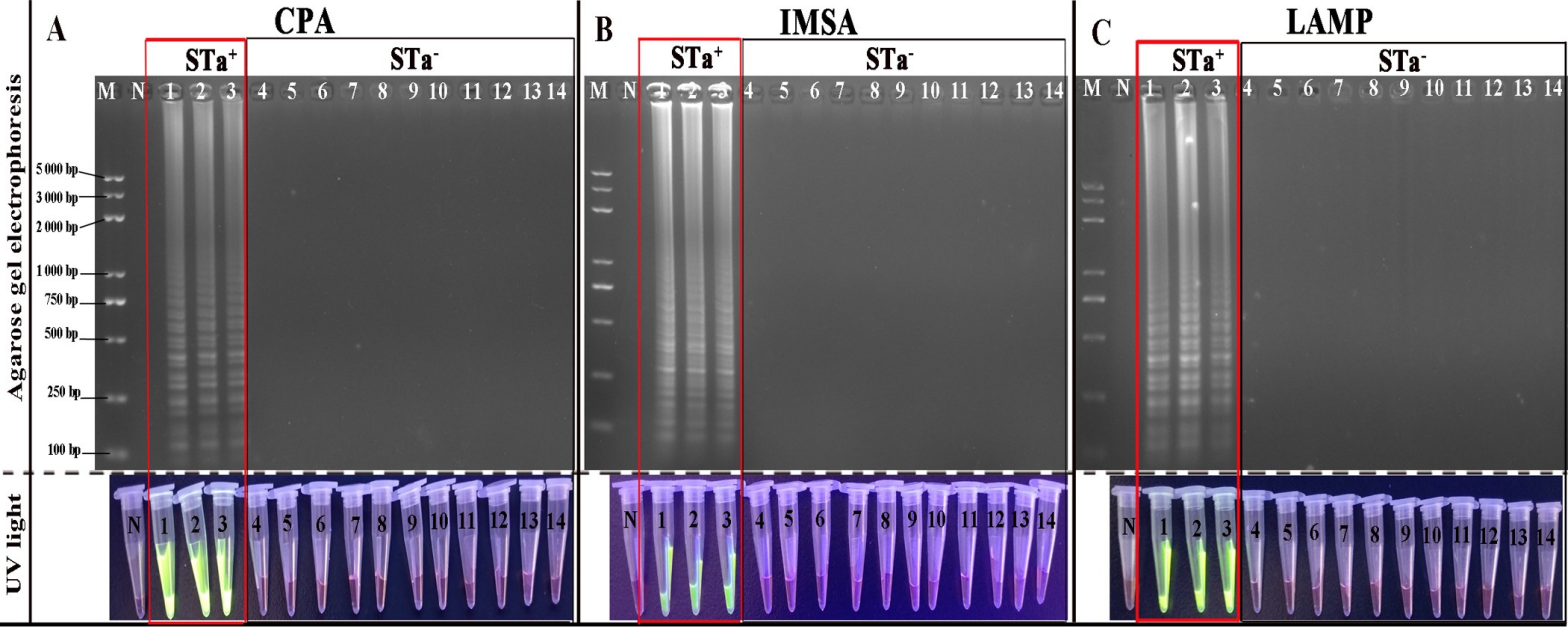
Specificity assessment of the CPA, IMSA and LAMP assays. A-C. Amplification products of CPA, IMSA and LAMP assays under UV light with agarose gel electrophoresis, respectively. M. Standard DNA molecule marker; N: negative control. 1–14: ATCC 35401, C83920, SD-12-01, C83915, O157: H7, C44498, SD-02-01, SD-17-03, ATCC 7966, ATCC 19115, ATCC 13076, ATCC 25923, ATCC 23715 and ATCC 27519, respectively. (The identity and source of each reference strain are listed in Table 1. All positive amplification results are indicated by red squares.).

Specificity of the freshly developed CPA and IMSA assays was compared with a LAMP assay, in which 8 strains of *E*. *coli* and 6 of other pathogenic bacteria, including *Salmonella*, *S*. *aureus* and *P*. *aeruginosa etc*. were tested. The results of agarose electrophoresis demonstrated that only the 3 strains of *E*. *coli* containing the STa gene tested positive, while 11 of the other pathogens gave negative results with no cross-reaction. This analysis using 8 *E*. *coli* pathogens also demonstrated that the CPA and IMSA primers designed for this study were located in the conserved regions of the STa genome with no cross-reaction with other non-STa^+^ *E*. *coli* strains (Fig 4).

### Sensitivity of the assays

**Fig 5:**
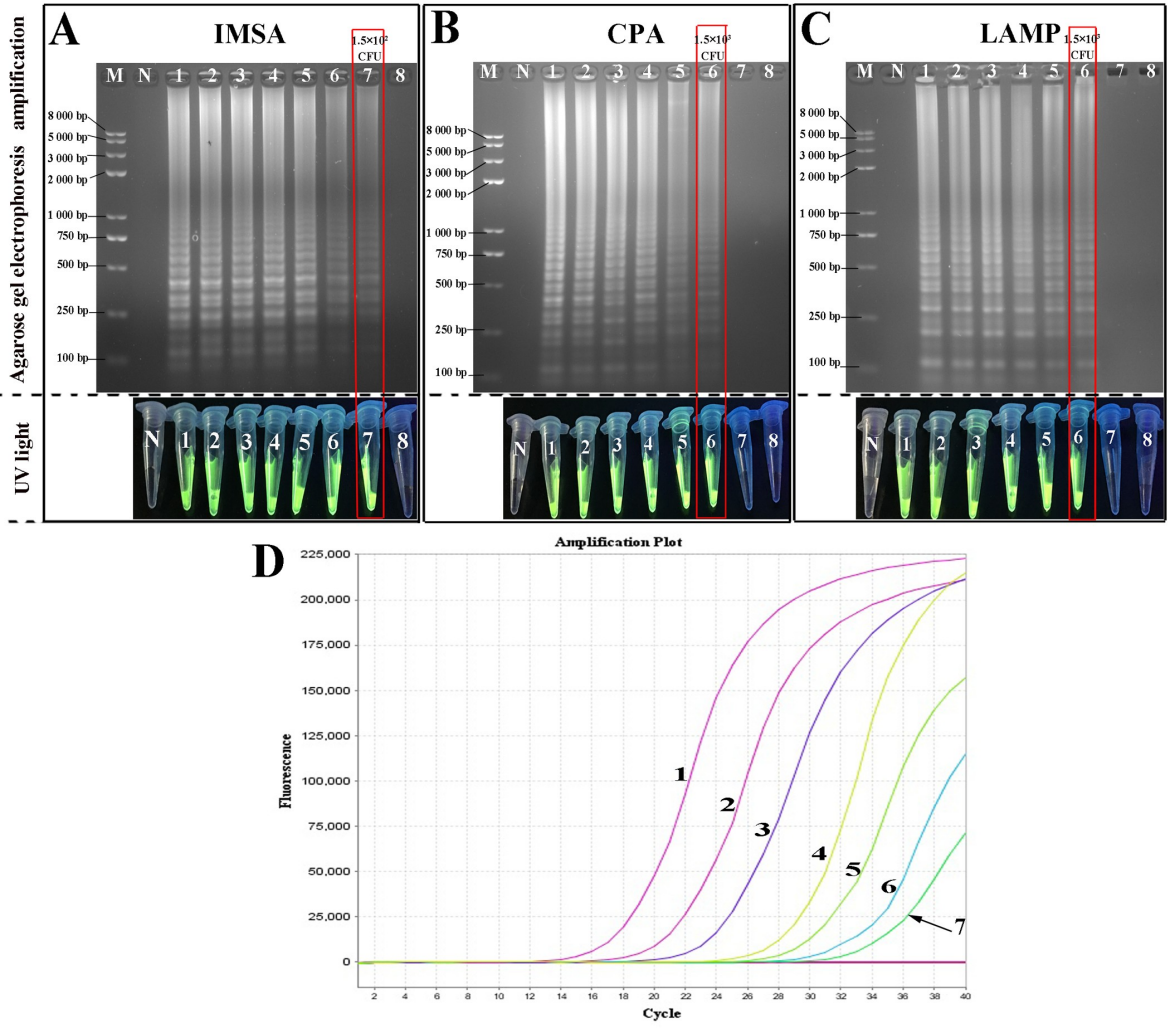
Sensitivity assessment of the IMSA, CPA, LAMP and qPCR assays. A-C. Amplification products from IMSA, CPA and LAMP assays under UV light and agarose gel electrophoresis, respectively. D. Analysis of limits of detection by qPCR. 1–8: 1.5×10^8^, 1.5×10^7^, 1.5×10^6^, 1.5×10^5^, 1.5×10^4^, 1.5×10^3^, 1.5×10^2^, 1.5×10^1^ CFU/mL, respectively. M: Standard DNA molecule marker. N: negative control. (The detection limits of the IMSA, CPA and LAMP assays are denoted with red squares.).

The limits of detection (LOD) of the CPA and IMSA assays were compared with real-time PCR, using a 10-fold serial dilution of DNA sample prepared from C83920 as the template with an initial concentration of 1.5×10^8^ CFU/reaction. Typical ladder-like patterns were obtained using as little as 1.5×10^2^ CFU of target DNA for IMSA and real-time PCR, while 1.5×10^3^ CFU/reaction were required the for the CPA and LAMP assays, respectively (Fig 5). The results indicate that the sensitivity of the CPA assay was weaker than that of the IMSA and real-time PCR assays.

### Use of CPA and IMSA to measure clinical specimens

**Fig 6:**
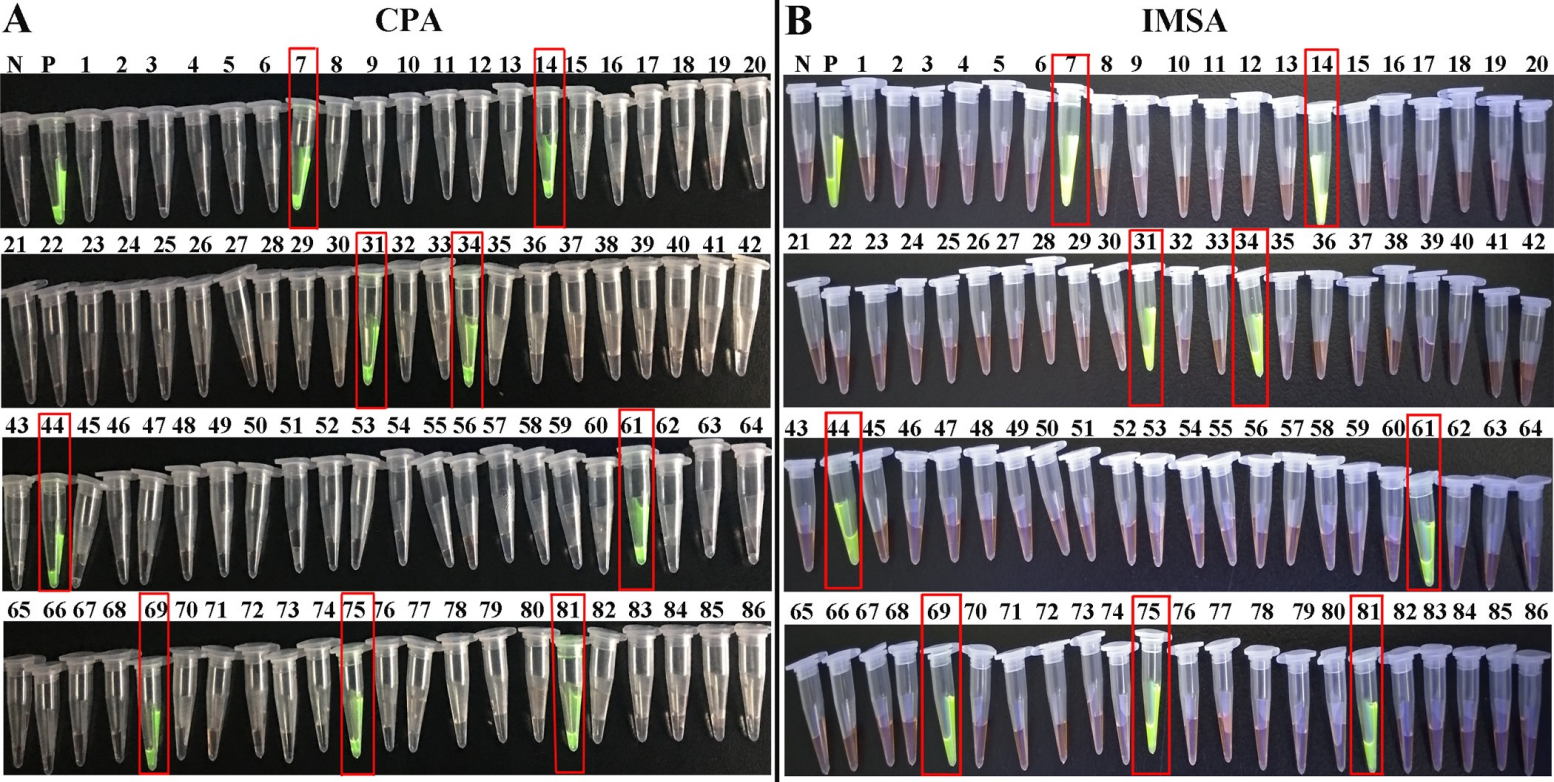
Clinical sample assessment of CPA and IMSA assays. A-B. Results of clinical samples assessed using the CPA and IMSA assays, respectively. N. Negative control; P. STa^+^ positive control. 1–86: clinical diarrhea specimens. (Positive amplification results are indicated by red squares.).

To evaluate the effectiveness of the CPA and IMSA assays to detect STa in clinical diarrhea specimens, a total of 86 *E*. *coli* strains were isolated from 265 suspected *E*. *coli* diarrhea samples by traditional bacterial isolation procedures, culture and biochemical assays. After mixing with SYBR Green I dye (Fig 6), observation under ultraviolet light demonstrated a positive detection rate for CPA and IMSA assays of 10.47% (9/86), which showed 100% agreement with the measurement results of the LAMP and real-time PCR assays (Table 3). The results indicated that development of the CPA and IMSA techniques have value in clinical applications and could be used as rapid detection technology for the STa gene.

**Table 3:** Detection results of clinical diarrhea samples.

Detection methods	Diarrheal specimens (n = 86)	
	Positive (%)	Negative (%)
CPA	9 (10.47)	77 (89.53)
IMSA	9 (10.47)	77 (89.53)
LAMP	9 (10.47)	77 (89.53)
Real-time PCR	9 (10.47)	77 (89.53)

## Discussion

Previous studies have shown that the vast majority of piglet diarrhea is caused by enterotoxigenic *Escherichia coli* (ETEC), resulting in huge financial losses in the agricultural sector in developing countries [27]. With the continuous development of isothermal amplification technology in recent years [28], additional new methods have been proposed to detect diarrhea caused by ETEC due to the advantages of rapidity, accuracy and low cost. In recent studies, Liu *et al*. reported the development of Loop-mediated isothermal amplification (LAMP), cross-priming amplification (CPA) and isothermal multiple self-matching initiated amplification (IMSA) to detect heat-labile enterotoxin-producing *Escherichia coli*. The three isothermal amplification assays demonstrated high specificity and sensitivity when detecting heat-labile enterotoxin-producing *E*. *coli* in clinical samples [24, 28]. Additionally, research by Yang, *et al*. also confirmed that IMSA demonstrated higher sensitivity than LAMP or CPA assays [19]. However, compared with other pathogenic bacteria, ETEC is more serotyped and relatively complicated. The homology between different enterotoxins is poor. It is difficult to design specific primers to detect all types of enterotoxin. STa enterotoxin is frequently identified in ETEC strains isolated from diarrhea in weaned piglets at a high rate of isolation, which indicates that veterinarians should pay more attention to the detection of STa toxin [6]. The aim of the present study was to develop and modify two rapid, accurate and sensitive isothermal amplification methods (IMSA, CPA) for the identification of heat-stable I enterotoxin (STa)-producing *E*. *coli*, and to provide a greater range of options for the detection of ETEC.

To avoid cross-contamination, the study was housed in leak-proof plastic devices (isothermal amplification tubes, IAT), the specific amplified products detected without opening the caps, preventing the possibility of amplicon cross-contamination and reducing the risk of false-positive results. No detection systems evaluated required opening of the tube to add SYBR Green I and so avoided the high risk of workspace contamination during amplification. Compared with a vertical flow visualization assay, this methodology has the advantages of simplicity and low cost, *etc*. In the current study, both methods relied on bst polymerase catalysis to achieve amplification within 1 h using specific primers without the need for an initial denaturation step. After optimization, both the CPA and the IMSA assays demonstrated high specificity, in which the ETEC strain carrying STa could be identified accurately among multiple different species, in comparison with the LAMP assay. In reference to sensitivity, the IMSA method is more sensitive than CPA, LAMP or real-time PCR, possibly related to the number of specific primers and regions on the recognition target sequences. Even if this makes the IMSA method applicable to the detection of fewer copies of STa strains in clinical samples, an increased number of primers will cause the method itself to produce false negative results or mismatched tolerance. Therefore, a large number of clinical diarrhea samples was collected and identified to evaluate the practicability and efficiency of the CPA and IMSA methods for clinical detection. In 86 clinical samples analyzed by CPA and IMSA, the results were compared with biochemical analysis and the results of LAMP and real-time PCR, with 9 positive reactions identified. All three isothermal amplification methods exhibited no false-negative or false-positive results, with sensitivity similar to that of real-time PCR. The evidence above suggests that the established IMSA method appears to have higher sensitivity, consistent with previous studies, and so can be used clinically as potential new technology for the detection of STa-producing ETEC strains.

In conclusion, for the first time two new isothermal amplification technologies for rapid identification of the STa gene, a common pathogen responsible for an economically important disease of livestock, have been developed and evaluated. These methods were found to be a potential rapid and sensitive tool for clinical analysis that can be used for diagnosis in the field or in small veterinary diagnosis units.

## Supporting information

S1 Raw images (PDF)
