# Supplementary material for: Establishment and application of isothermal amplification techniques for the detection of heat-stable I enterotoxin of enterotoxigenic Escherichia coli
Source: PLoS One. 2020 Apr 21;15(4):e0230881. doi: 10.1371/journal.pone.0230881 (PMC7173923; doi:10.1371/journal.pone.0230881)
Supplement: S1 Raw images — (PDF) [file pone.0230881.s001.pdf]

Fig.2

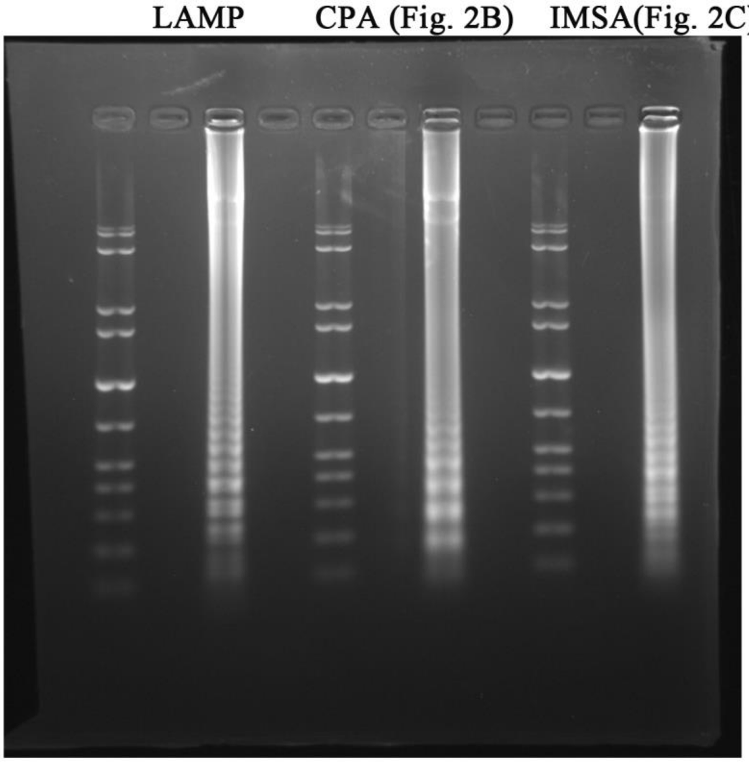

The figure above is the primers verification results of LAMP, CPA and IMSA methods. The middle and right parts are CPA (Fig.2B) and IMSA (Fig.2C) respectively, and the images were taken in gel imaging system.

Fig. 3B

(CPA)

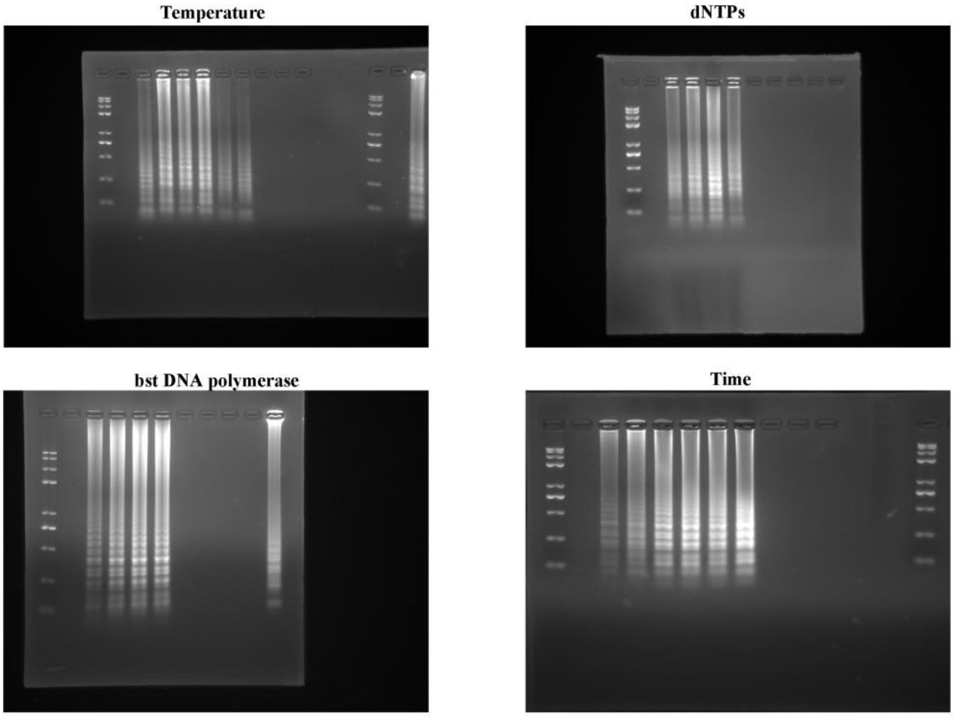

The above 4 agarose amplification photograph are taken by Fig. 3B. The images are taken by gel imaging system.

Fig. 3C

(IMSA)

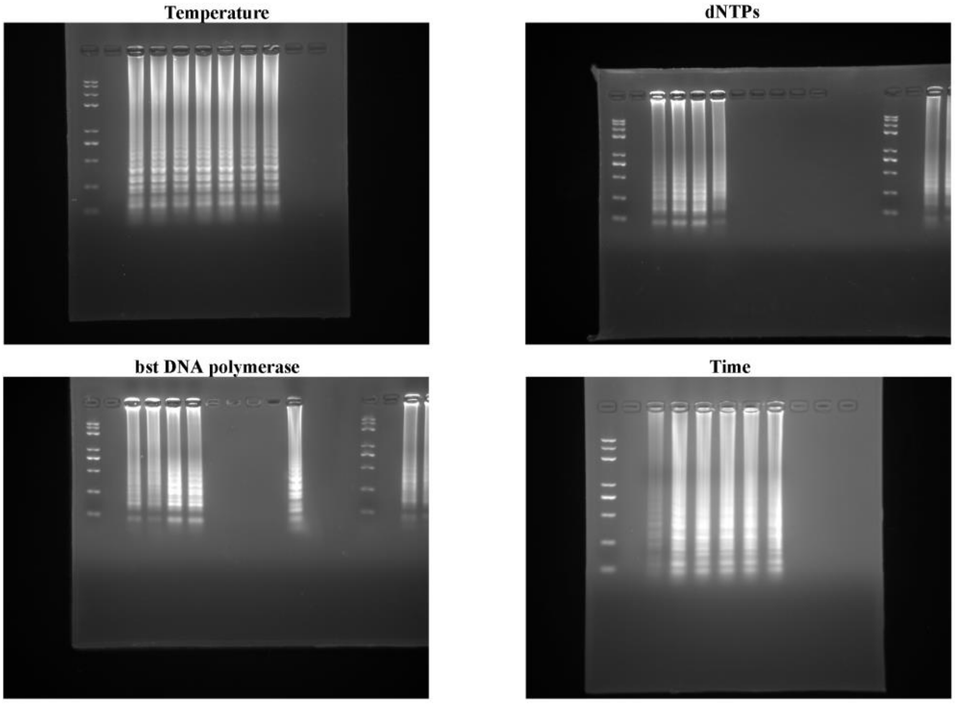

The above 4 agarose amplification photograph are taken by Fig. 3C. The images are taken by gel imaging system.

Fig. 4A

(CPA)

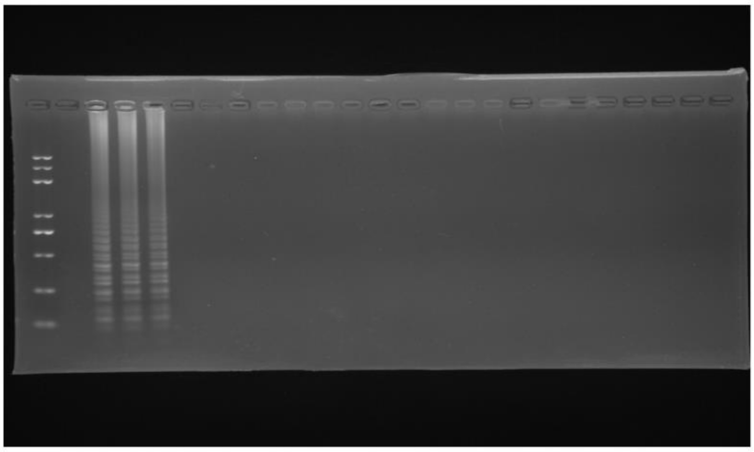

The above agarose amplification photograph is the result of CPA specificity assay and taken by gel imaging system.

Fig. 4B

(IMSA)

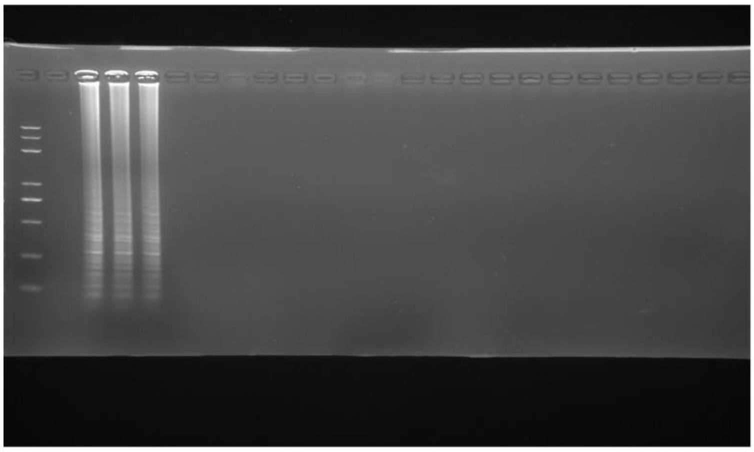

The above agarose amplification photograph is the result of IMSA specificity assay and taken by gel imaging system.

Fig. 4C

(LAMP)

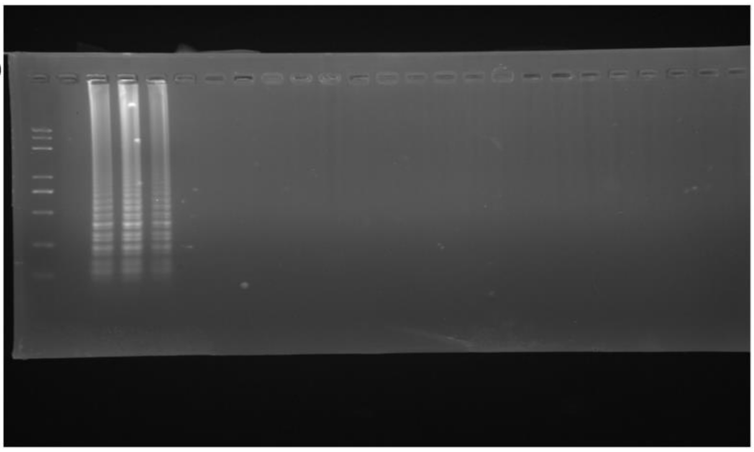

The above agarose amplification photograph is the result of LAMP specificity assay and taken by gel imaging system.

Fig. 5A
(IMSA)

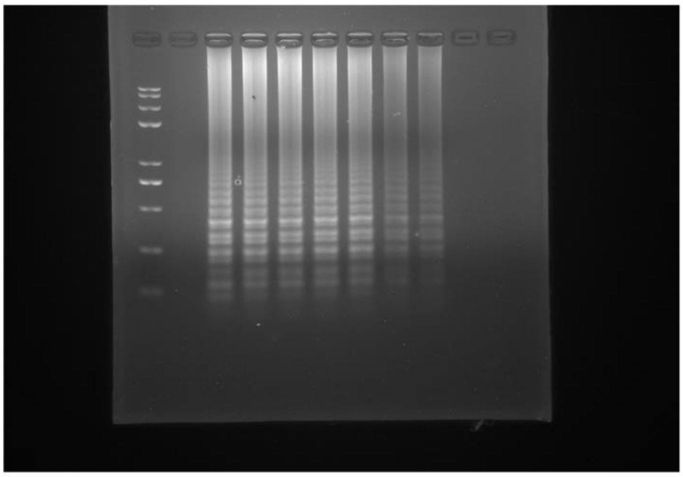

The above agarose amplification photograph is the result of IMSA sensitivity assay and taken by gel imaging systen

Fig. 5B
(CPA)

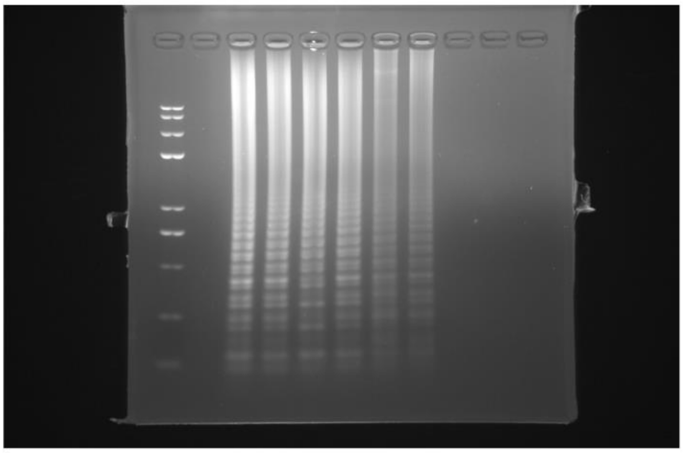

The above agarose amplification photograph is the result of CPA sensitivity assay and taken by gel imaging system.

Fig. 5C
(LAMP)

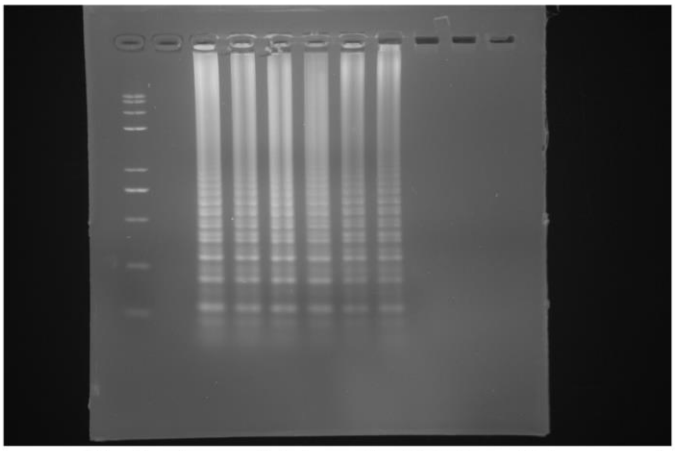

The above agarose amplification photograph is the result of LAMP sensitivity assay and taken by gel imaging system.
